# γ-Enolase enhances Trk endosomal trafficking and promotes neurite outgrowth in differentiated SH-SY5Y cells

**DOI:** 10.1186/s12964-021-00784-1

**Published:** 2021-12-11

**Authors:** Anja Pišlar, Janko Kos

**Affiliations:** 1 Department of Pharmaceutical Biology, Faculty of Pharmacy, University of Ljubljana, Aškerčeva 7, 1000 Ljubljana, Slovenia; 2 Department of Biotechnology, Jožef Stefan Institute, Jamova 39, 1000 Ljubljana, Slovenia

**Keywords:** γ-Enolase, Trk receptor, Clathrin-mediated endocytosis, Late endosomes, Neurite outgrowth

## Abstract

**Background:**

Neurotrophins can activate multiple signalling pathways in neuronal cells through binding to their cognate receptors, leading to neurotrophic processes such as cell survival and differentiation. γ-Enolase has been shown to have a neurotrophic activity that depends on its translocation towards the plasma membrane by the scaffold protein γ1-syntrophin. The association of γ-enolase with its membrane receptor or other binding partners at the plasma membrane remains unknown.

**Methods:**

In the present study, we used immunoprecipitation and immunofluorescence to show that γ-enolase associates with the intracellular domain of the tropomyosin receptor kinase (Trk) family of tyrosine kinase receptors at the plasma membrane of differentiated SH-SY5Y cells.

**Results:**

In differentiated SH-SY5Y cells with reduced expression of γ1-syntrophin, the association of γ-enolase with the Trk receptor was diminished due to impaired translocation of γ-enolase towards the plasma membrane or impaired Trk activity. Treatment of differentiated SH-SY5Y cells with a γ-Eno peptide that mimics γ-enolase neurotrophic activity promoted Trk receptor internalisation and endosomal trafficking, as defined by reduced levels of Trk in clathrin-coated vesicles and increased levels in late endosomes. In this way, γ-enolase triggers Rap1 activation, which is required for neurotrophic activity of γ-enolase. Additionally, the inhibition of Trk kinase activity by K252a revealed that increased SH-SY5Y cell survival and neurite outgrowth mediated by the γ-Eno peptide through activation of signalling cascade depends on Trk kinase activity.

**Conclusions:**

These data therefore establish the Trk receptor as a binding partner of γ-enolase, whereby Trk endosomal trafficking is promoted by γ-Eno peptide to mediate its neurotrophic signalling.

**Graphical Abstract:**

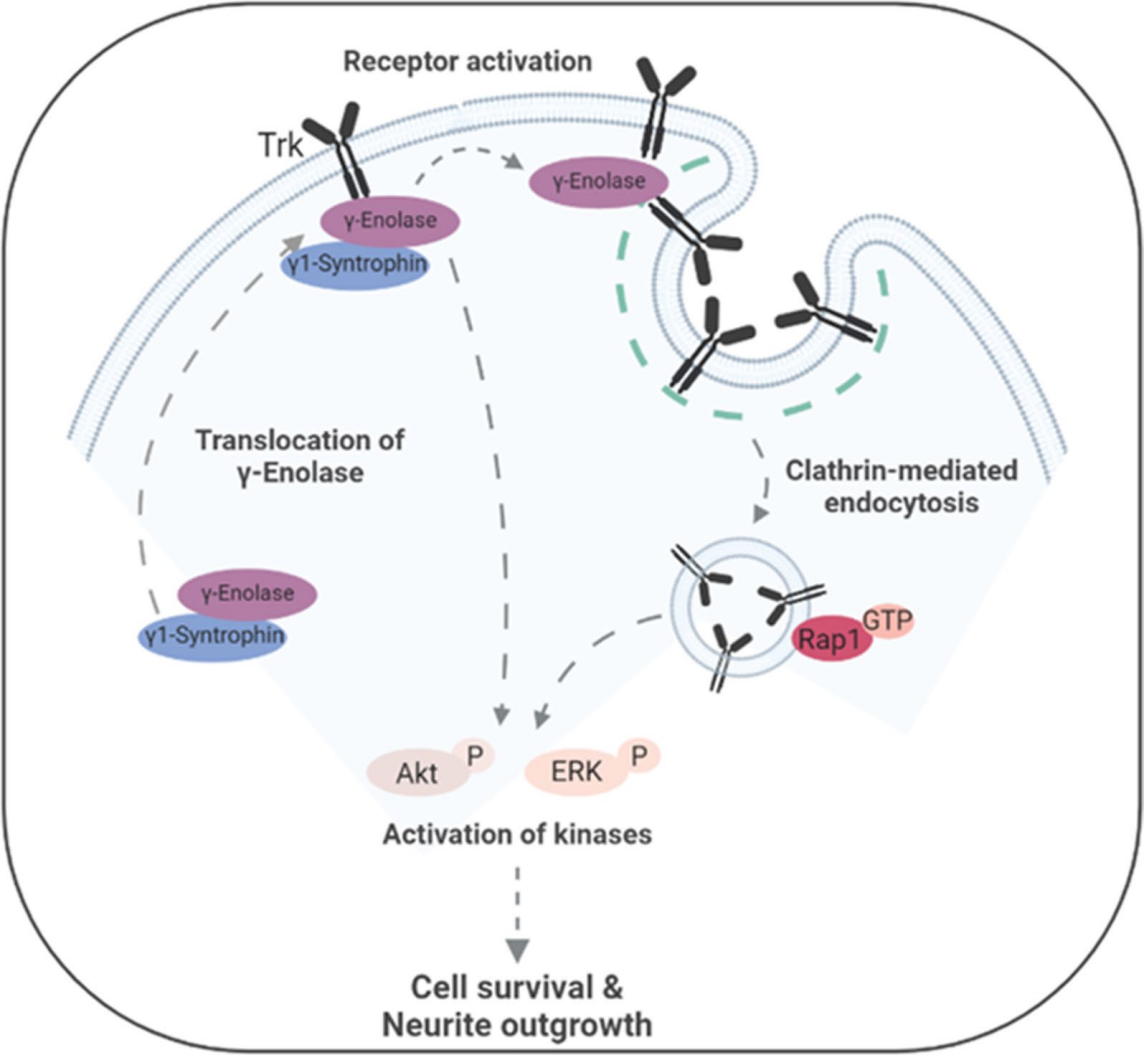

**Video abstract**

**Supplementary Information:**

The online version contains supplementary material available at 10.1186/s12964-021-00784-1.

## Background

Maintenance of the neuronal network system results in an orchestrated pool of events that involve neuronal cell migration through formation and extension of axons and dendrites, which leads to neurite outgrowth and axon guidance [1, 2]. Neurotrophic factors have important roles in these processes, with the control of nerve growth, cell differentiation, regeneration and survival, which include the neurotrophins nerve growth factor (NGF), brain-derived neurotrophic factor (BDNF), neurotrophin-3 (NT-3) and neurotrophin-4/5 (NT-4/5) [3–5]. As well as these established nerve growth factors, several other proteins have been suggested to have neurotrophic activities, among which γ-enolase has been defined as a neurotrophic-like factor [6–8].

Enolase is a glycolytic enzyme that forms homodimers and heterodimers composed of α, β and γ subunits [9]. In mammalian brain, there are three enolase isoforms: αα, αγ and γγ [10]. The αγ and γγ isoforms are also known as neuron-specific enolase, and they are found primarily in tissues of the central nervous system, localised to neurons and neuroendocrine cells [11]*.* Previous studies have shown that the C-terminal part of neuron-specific enolase has a neurotrophic activity at the plasma membrane of neuronal cells [7, 8, 12, 13]. Indeed, γ-enolase promotes neuronal survival and differentiation, and neurite regeneration through activation of the phosphatidylinositol 3-kinase (PI3K) and mitogen-activated protein kinase (MAPK) signal transduction pathways. This activation results in regulation of the downstream molecular processes of cytoskeleton reorganisation and cell remodelling, with activation of transcription factors and regulation of the cell cycle [6]. Although the intracellular trafficking of cytosolic γ-enolase towards the plasma membrane is known to be regulated by scaffold protein γ1-syntrophin [14], the mechanism of its coupling with its membrane receptor or with other proteins downstream of this signalling pathway remains unknown.

The biological effects of neurotrophins on neuronal cells are mediated by two classes of specific membrane receptors. The first of these, neurotrophin receptor p75 (p75^NTR^), belongs to the family of death receptors. p75^NTR^ does not show any binding specificity for the different neurotrophins, and it is involved in the regulation of neuron survival through its induction of apoptosis [15–17]. The second class of these receptors, the tropomyosin receptor kinase (Trk) family of tyrosine kinase receptors, binds neurotrophins in a specific manner. TrkA is predominantly activated by NGF, TrkB by BDNF and NT-4/5, and TrkC by NT-3 [18]. Neurotrophin binding to the Trk receptor induces receptor dimerisation and phosphorylation of tyrosine residues that function as docking sites for adaptor proteins. Once associated with adaptor proteins, Trk can activate signalling pathways that are important for its cellular activities. In this way, neurotrophin signalling through Trk receptors regulates cell proliferation and survival, and axon and dendrite outgrowth [19, 20].

In our previous study, we showed regulation of the neuroprotective role of γ-enolase by interference with the p75^NTR^ receptor and its downstream signalling in neuronal cells treated with the amyloid-β-peptide [21]. However, under non-degenerative conditions, there was no interaction of γ-enolase with the p75^NTR^ receptor. Therefore, in the present study, we investigated the Trk receptor as the focus of γ-enolase neurotrophic signalling. Using immunoprecipitation and confocal microscopy, we demonstrate here the association of γ-enolase with Trk in neuroblastoma SH-SY5Y cells. Moreover, we define the involvement of γ-enolase in Trk internalisation and the consequent receptor trafficking to late endosomes that leads to neurite outgrowth.

## Materials and methods

### Reagents

The C-terminal 30-amino-acid sequence of human brain γ-enolase was synthesised by Biosynthesis (Lewisville, TX, USA), here defined as the γ-Eno peptide (AKYNQLMRIEEELGDEARFAGHNFRNPSVL). The inhibitor of clathrin-mediated endocytosis, chlorpromazine (CPZ), and the inhibitor of protein tyrosine kinase activity of the Trk family, K252a, were from Sigma-Aldrich (St. Louis, MO, USA). The Rap1-processing inhibitor, GGTI-298, was from Calbiochem (Millipore, Billerica, MA, USA). The MEK5 catalytic activity inhibitor, BIX02189, was from Santa Cruz Biotechnology (Santa Cruz, CA, USA). All of these inhibitors were dissolved in dimethyl sulphoxide (Sigma-Aldrich), the final concentration of which never exceeded 0.01% (v/v). The goat polyclonal antibodies against γ1-syntrophin, the extracellular domain of Trk A and the N-terminal of γ-enolase, and the mouse monoclonal antibody against the C-terminal of γ-enolase, were from Santa Cruz Biotechnology. The rabbit monoclonal antibodies to detect endogenous levels of total Trk protein (*pan*-Trk) and endogenous levels of p75^NTR^ were from Cell Signaling Technology (Danvers, MA, USA). The rabbit polyclonal antibodies against *pan*-cadherin and MAP2, and the mouse monoclonal antibody against clathrin heavy chain were from Abcam (London, UK). The rabbit polyclonal antibody against Rab7 was from Sigma-Aldrich. All of the secondary antibodies conjugated with Alexa Fluor dyes were from Molecular Probes (Thermo Fisher Scientific, Waltham, MA, USA). The probe for detection of filamentous actin phalloidin conjugated with tetramethylrhodamine B isothiocyanate (TRITC) was from Sigma-Aldrich. All of the other reagents were of the highest grade possible and were from standard commercial sources.

### Cell culture and treatments

Human neuroblastoma SH-SY5Y cells were obtained from American Type Culture Collection (CRL-2266; Manassas, VA, USA). The cells were cultured in complete growth medium of advanced Dulbecco’s modified Eagle’s medium (Gibco, Thermo Fisher Scientific) supplemented with 10% foetal bovine serum (HyClone, Logan, UT, USA), 2 mM L-glutamine, 50 U/mL penicillin and 50 μg/mL streptomycin (Sigma-Aldrich). The cells were maintained at 37 °C in a humidified atmosphere of 95% air and 5% CO_2_, and grown to 80% confluence.

Prior to the experiments, the complete growth medium was replaced with serum-free medium for 24 h, unless otherwise indicated. The γ-Eno peptide treatments were carried out in serum-free medium with 100 nM γ-Eno peptide for the times indicated. The CPZ treatments were at 1 µM for the times indicated or 2 h prior to γ-Eno peptide treatment. The treatments with 50 nM to 500 nM K252a and 5 µM GGTI-298 were carried out in serum-free medium 1 h prior to γ-Eno peptide treatment.

### γ1-Syntrophin silencing

Silencing of γ1-syntrophin was performed using the Stealth RNAi/γ1-Syn system. The single stranded oligonucleotides used to target the γ1-syntrophin mRNA were (RNA)-GGUAGCUUGUUUGGACCCUCUAUUU and (RNA)-AAAUAGAGGGUCCAAACAAGCUACC (Invitrogen, Carlsbad, CA, USA). The SH-SY5Y cells were transiently transfected with Stealth RNAi/γ1-Syn using Lipofectamine 2000 (Invitrogen, Thermo Fisher Scientific), according to the manufacturer protocol. After 24 h or 48 h of transfection, the silencing of γ1-syntrophin was determined by flow cytometry using a goat polyclonal antibody against γ1-syntrophin (Additional file 1: Figure S1).

### Immunoprecipitation and Western blot analysis

For immunoprectipitation, the SH-SY5Y cells were seeded in complete medium in 25-cm^2^ culture flasks (5 × 10^6^/well), and after serum deprivation of the wild-type and transfected SH-SY5Y cells for 24 h or treated with K252a as indicated, they were harvested in cell lysis buffer (50 mM HEPES, pH 6.5, 150 mM NaCl, 1 mM EDTA, 1% Triton X-100), supplemented with a cocktail of protease and phosphatase inhibitors, and incubated for 30 min on ice. For detection of signalling kinases in cell lysates, cells were seeded in 6-well plates (1 × 10^6^/well). After treatment as indicated, cells were harvested in cell lysis buffer (50 mM HPES, pH 6.5, 150 mM NaCl, 1 mM EDTA, 1% Triton X-100) supplemented with a cocktail of protease and phosphatase inhibitors, and incubated for 30 min on ice. Protein determinations and Western blotting were performed as described previously [6].

For immunoprecipitation, the protein concentrations were adjusted by addition of lysis buffer, taken to pH 8.0 with 1 M NaOH, and to a final concentration of 100 µg protein in 50 µL. Then 5 µg rabbit anti-*pan*-Trk antibody or rabbit IgG antibody (Santa Cruz Biotechnology) were added, with an overnight incubation at 4 °C. To purify the immune complexes, 50 µL of this mixture was added to pre-washed protein A–Sepharose beads (Amersham Pharmacia Biotech AB, Uppsala, Sweden) and mixed for an additional 2 h at 4 °C. The beads were then washed with binding buffer (140 mM phosphate buffer, pH 8.2), and the immune complexes were eluted by boiling in sodium dodecyl sulphate sample buffer. To detect the immune complexes, a mouse anti-γ-enolase antibody (1:500; Santa Cruz Biotechnology) was used in the Western blotting, followed by incubation with horse radish peroxidase-conjugated anti-mouse antibodies (1:5000; Millipore). The signals were visualised using enhanced chemiluminiscence detection kit (Thermo Fisher Scientific). The band intensities were quantified using the GeneTools software (Syngene, Cambridge, UK), and are expressed relative to those of the wild-type IgG.

For western blot, the following primary antibodies were applied: mouse anti-γ-enolase (1:500; Santa Cruz Biotechnology), rabbit anti-phospho-ERK1/2 (1:2000; Cell Signaling), rabbit anti-ERK1 (1:1000; Santa Cruz Biotechnology), rabbit anti-ERK2 (1:5000; Santa Cruz Biotechnology), rabbit anti-phospho-Akt (1:1000; Cell Signaling), rabbit anti-Akt (1:1000; Cell Signaling), mouse-anti-phospho-Trk (1:200; Santa Cruz Biotechnology), and mouse anti-β-actin (1:500; Sigma). Signals from anti-rabbit or anti-mouse HRP-conjugated secondary antibodies (1:5000; Millipore) were visualized with an enhanced chemiluminescence detection kit (Thermo Fisher Scientific). The band intensities were quantified using Gene Tools software (Sygene), and expressed as values relative to those of controls.

### Double immunofluorescence staining

The SH-SY5Y cells were seeded in complete medium on glass coverslips in 24-well culture plates (2 × 10^4^/mL) in duplicate, and the next day they were transfected and/or differentiated by serum deprivation, as indicated above. The cells were then fixed with 5% formalin in phosphate-buffered saline (PBS), pH 7.4, for 30 min at room temperature, and then permeabilised with 0.5% Tween 20 in PBS for 10 min. Non-specific staining was blocked with 3% bovine serum albumin (BSA) in PBS for 30 min at room temperature. The cells were then incubated with 10 µg/mL mouse anti-γ-enolase, 5 µg/mL rabbit anti-*pan*-Trk, 5 µg/mL rabbit anti-p75^NTR^, 6 µg/mL mouse anti-clathrin, 10 µg/mL goat anti-γ-enolase, 2 µg/mL rabbit anti-cadherin, 10 µg/mL goat anti-Trk or 5 µg/mL rabbit anti-Rab7 antibodies in blocking buffer for 2 h at room temperature. Afterwards, the cells were washed with PBS and further incubated with Alexa Fluor 488- and Alexa Fluor 555-labelled secondary antibodies (1:1000), for an additional 1.5 h. After washing with PBS, ProLong Antifade kit (Molecular Probes) was used for mounting the coverslips on glass slides. Fluorescence microscopy was performed using a confocal microscope (LSM 710; Carl Zeiss, Oberkochen, Germany) with the ZEN 2011 image software. The relative co-localisation areas were analysed for several cells (≥ 10). The quantification of co-localisation areas is represented by the mean number of pixels in the third quadrant in the scatter plots for two fluorescence intensities.

### Flow cytometry analysis of Trk cell surface expression

The SH-SY5Y cells were seeded in complete medium in 12-well culture plates (2 × 10^5^/mL) in duplicate, and the next day they were treated with γ-Eno peptide in the absence and presence of CPZ, as described. After the times indicated, the cells were harvested and washed with ice-cold PBS, pH 7.4, and further with 3% BSA in PBS, containing 1% sodium azide. Non-specific staining was blocked with 3% BSA in PBS for 20 min at room temperature. The cells were then incubated with antibodies against the extracellular domain of TrkA (4 µg/mL) for 45 min at 4 °C, washed with PBS, and incubated with the Alexa Fluor 555-labelled secondary antibody (1:500) for 45 min at room temperature. After washing with PBS, the cells were fixed with 5% formalin in PBS. Finally, the cells were washed with PBS and analysed by flow cytometry (FACS Calibur; BD Biosciences). The data were analysed using the FlowJo software (Tree Star Inc., Ashland, OR, USA), and are expressed relative to the untreated (Control) cells.

### Cell viability assay

Cell viabilities were evaluated using the [3-(4,5-dimethylthiazol-2-yl)-5-(3-carboxymethoxyphenyl)-2-(4-sulfophenyl)-2H-tetrazolium inner salt (MTS) assay. The SH-SY5Y cells were seeded in complete medium in 96-well culture plates (2 × 10^4^/well), in quadruplicate. The next day, they were treated as described above. Following the 48-h treatments, the cell viabilities were determined using CellTiter 96 Aqueous One Solution cell proliferation assay (Promega, Madison, WI, USA), according to the manufacturer instructions. Absorbance was measured with an automatic microplate reader (Safire^2^; Tecan, Männedorf, Switzerland) at 490 nm. The data are expressed relative to the untreated (Control) cells.

### Evaluation of neurite outgrowth

The SH-SY5Y cells were seeded in complete medium in 24-well culture plates (5 × 10^4^/well) in duplicate. The next day, they were treated as described above. Following the 48 h treatments, neurite outgrowth was evaluated by morphological examination under a motorised inverted microscope (Nikon TMS, Tokio, Japan). Cells with one or more neurites where the length was twice the diameter of the cell body were scored as positive. The cells were observed using EVOS Cell Imaging System (Thermo Fisher Scientific) and representative images of treatments with γ-Eno peptide in absence or presence of GGTI-298 or K252a inhibitors to support evaluation of neurite outgrowth are shown in Additional file 1: Figure S2. At least 100 cells were quantified for each group in each experiment. The data are expressed relative to the untreated (Control) cells.

### Determination of cell F-actin content

The SH-SY5Y cells were seeded in complete medium in 24-well culture plates (5 × 10^4^/well) in duplicate. The next day, they were treated as described above. Following the 2 h treatments, the cells were washed with PBS, pH 7.4, and incubated with 500 ng/mL phalloidin-TRITC conjugate in serum-free medium for 30 min at 37 °C. The cells were then washed with PBS and the cell F-actin content was determined using a flow cytometer (FACS Calibur; BD Biosciences) by gating living cells based on their forward and side scatter. The relative F-actin contents of the cells were evaluated using the FlowJo software, as proportional to the relative mean fluorescence intensity, which was determined as the ratio of the mean fluorescence of each sample relative to the fluorescence intensity of non-treated cells.

For the fluorescence imaging of cell F-actin, the SH-SY5Y cells were seeded in complete medium on glass coverslips in 24-well culture plates (2 × 10^4^/mL) in duplicate. Following the 2-h treatments as described, the cells were fixed with 5% formalin in PBS, pH 7.4, supplemented with 1 mM MgCl_2_ and 0.1 mM CaCl_2_, for 5 min at room temperature, and then permeabilised with 0.1% Triton X-100 in PBS for 3 min. After washing with PBS, the cells were incubated with 500 ng/mL phalloidin-TRITC conjugate in 20 mM Tris, pH 7.4, for 30 min at 37 ºC. After additional washing with PBS, ProLong Antifade kit with DAPI (Molecular Probes) was used for mounting the coverslips on glass slides. Fluorescence microscopy was performed using a motorised inverted microscope (IX 81; Olympus Biosystems Gmbh, Munich, Germany) with the CellR image software (Olympus).

### Flow cytometry analysis of MAP2 expression

The SH-SY5Y cells were seeded in complete medium in 12-well culture plates (2 × 10^5^/mL) in duplicate. The next day, they were treated as described above. Following the 24-h treatments, the cells were washed with PBS, pH 7.4, fixed with 5% formalin for 15 min at room temperature, and further permeabilised with 0.5% Tween 20 in PBS for 10 min. Non-specific staining was blocked with 3% BSA in PBS for 30 min at room temperature. The cells were then incubated with an antibody against MAP2 (2.5 µg/mL) in blocking buffer for 45 min at 4 °C. After washing with PBS, the cells were incubated with Alexa Fluor 555-labelled secondary antibody (1:500) for an additional 30 min at room temperature. Finally, the cells were washed with PBS and analysed by flow cytometry (FACS Calibur; BD Biosciences). The data were analysed using the FlowJo software, and are expressed relative to the untreated (Control) cells.

### Statistical analysis

The results shown are representative of at least two independent experiments, each performed in at least duplicate, as means ± standard deviation (SD). Statistical analysis was performed by either two tailed *t* test to compare two groups or one-way analysis of variance (ANOVA) to compare three or more groups followed by Dunnett’s post hoc test (to compare γ-Eno-treated cells to untreated cells—Control) or Tukey’s post hoc test (to compare inhibitor-pretreated cells to γ-Eno-treated cells) using GraphPad Prism, version 6. A value of *P* < 0.05 was considered as the level of statistical significance.

## Results

### γ-Enolase associates with the intracellular domain of the Trk receptor at the plasma membrane

Translocation of γ-enolase towards the plasma membrane by the scaffold protein γ1-syntrophin is a pre-requisite for its neurotrophic activity [14]. This is mediated through activation of the PI3K and MAPK signal transduction pathways [6]. For these pathways, signalling through neurotrophin receptors is prerogative [22], which prompted us to search for γ-enolase-protein interactions at the plasma membrane. Initially, confocal immunofluorescence microscopy with the *pan*-specific Trk antibody of the SH-SY5Y cells indicated some co-localisation of γ-enolase with the intracellular domain of the neurotrophin receptor Trk, which was mainly seen in the peri-plasma membrane region (Additional file 1: Figure S3A). This co-localisation was also examined in SH-SY5Y cells differentiated to neuronal-*like* cells by serum deprivation. After 24 h of this differentiation, γ-enolase was highly concentrated at the plasma membrane region and showed increased co-localisation with Trk (Additional file 1: Figure S3A). Only weak co-localisation of γ-enolase with the other neurotrophin receptor p75^NTR^ was seen for the control (Additional file 1: Figure S3B) and differentiated SH-SY5Y cells, with none at the plasma membrane (Additional file 1: Figure S3B).

**Fig. 1:**
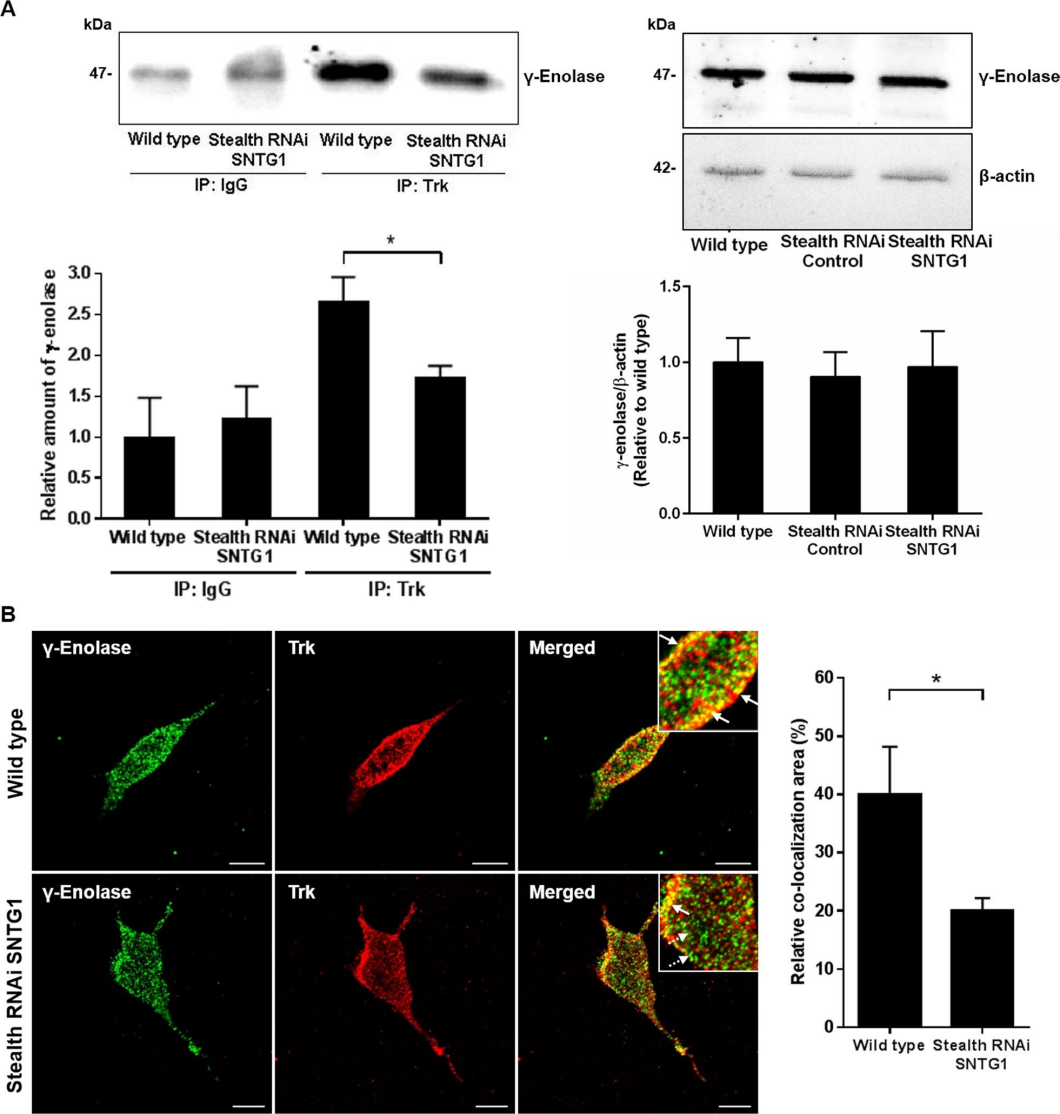
γ-Enolase associates with the intracellular domain of Trk in SH-SY5Y cells. **A** Representative co-immunoprecipitation (top) and quantification (bottom; as relative amount of γ-enolase in the immunoprecipitate in wild-type *vs.* transfected cells) of γ-enolase and Trk from wild-type and transfected SH-SY5Y cells with down-regulated γ1-syntrophin expression (Stealth RNAi SNTG1). Wild-type and transfected cells were differentiated by serum deprivation for 24 h, and then lysed and immunoprecipitated (IP) with either non-specific rabbit IgG or rabbit anti-*pan*-Trk antibodies (Trk). The immunoprecipitates (*left panel*) and lysates for immunoprecipitation (*right panel*) were analysed by Western blotting probed with a mouse anti-γ-enolase antibody. In case of lysates, β-actin was used as loading control. Data are means ± SD of two independent experiments (n = 2) (one-way ANOVA, Dunnett’s test, *P < 0.05) **B** Representative images (left) and quantification (right) of double immunofluorescence staining for γ-enolase (green fluorescence) and *pan*-Trk (red fluorescence) in wild-type and transfected SH-SY5Y cells 24 h after serum deprivation, showing stronger co-localization of γ-enolase with Trk (solid arrows) in wild type cells compared to transfected cells (dashed arrows). Data are means ± SD of the pixels in the third quadrant of scatter plot (cell numbers ≥ 10) (two tailed *t* test, * *P* ˂ 0.05). Scale bars: 10 µm

We then looked for direct association of γ-enolase with Trk at the plasma membrane using affinity immunoprecipitation assays. As shown in Fig. 1A, a *pan*-specific antibody for endogenous Trk co-immunoprecipitated γ-enolase from lysates of SH-SY5Y cells differentiated for 24 h. No effects were seen with the control IgG, which demonstrated specific binding between Trk and γ-enolase. There were no interactions between the control antibody and γ-enolase in transfected cells with decreased levels of γ1-syntrophin. However, the co-immunoprecipitation of Trk and γ-enolase was significantly lower in comparison to wild-type cells (Fig. 1A, *left panel*), wherein the level of γ-enolase expression did not significantly differ between wild type cells and cells with down-regulated γ1-syntrophin protein expression (Fig. 1A, *right panel*). This last observation was also confirmed by confocal immunofluorescence microscopy, where strong co-localisation of γ-enolase with the intracellular domain of Trk in the peri-plasma membrane region was diminished in differentiated SH-SY5Y cells with decreased γ1-syntrophin expression, in comparison with wild-type cells (Fig. 1B). This indicated that association of γ-enolase with the intracellular domain of Trk receptor at the plasma membrane is guided by γ1-syntrophin-mediated trafficking of γ-enolase.

### γ-Enolase is present in clathrin-coated vesicles

**Fig. 2:**
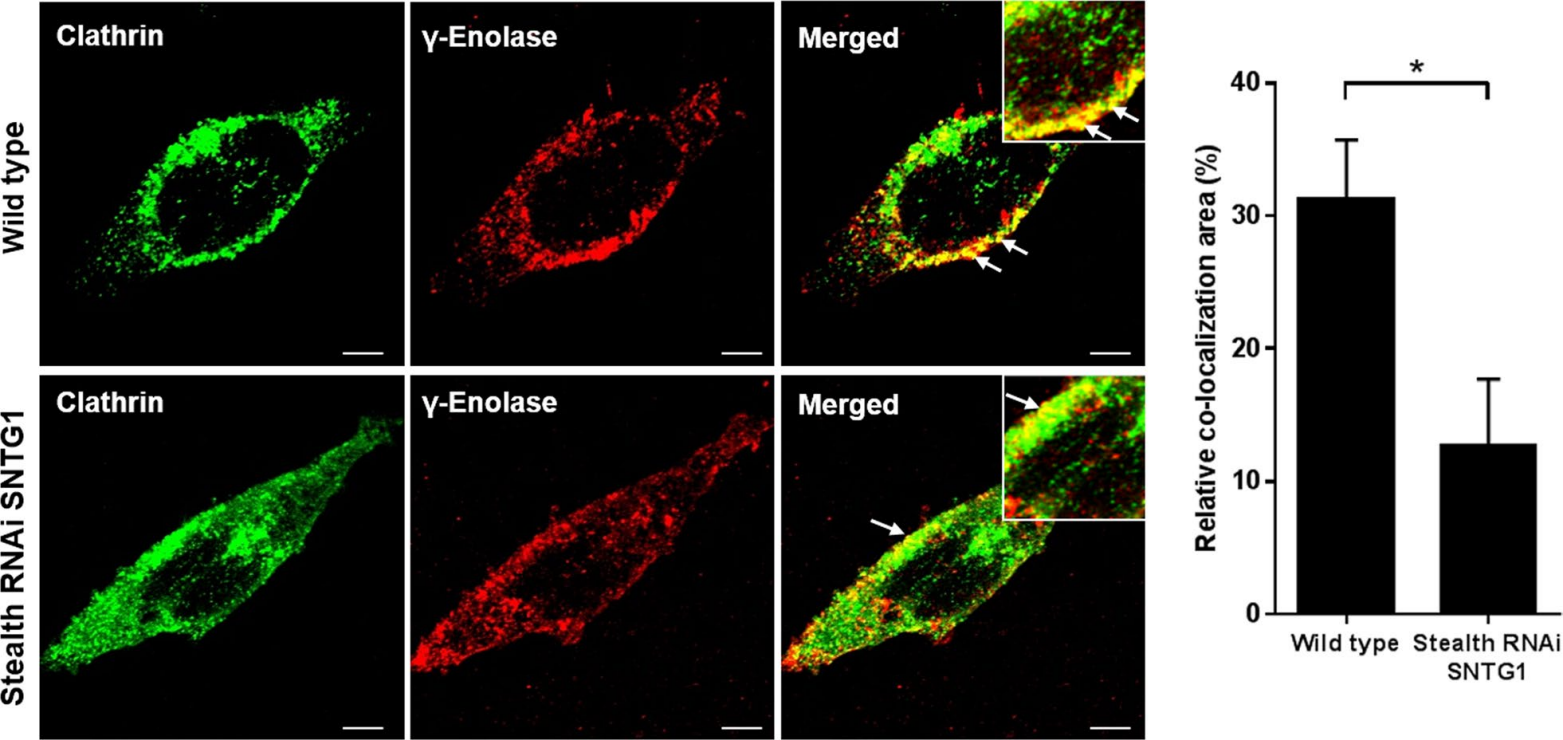
γ-Enolase localisation in clathrin-coated vesicles in SH-SY5Y cells. Representative images (left) and quantification (right; as relative co-localisation area of clathrin and γ-enolase) of double immunofluorescence staining for clathrin heavy chain (green fluorescence) and γ-enolase (red fluorescence) in wild-type and transfected SH-SY5Y cells with down-regulated γ1-syntrophin expression (Stealth RNAi SNTG1). Stronger co-localization of γ-enolase with clathrin heavy chain (solid arrows) was observed in wild type cells compared to transfected cells. Data are means ± SD of the pixels in the third quadrant of the scatter plot (cell numbers ≥ 10) (two tailed *t* test, * *P* ˂ 0.05). Scale bars: 10 µm

Internalisation and intracellular transport of neurotrophin receptor complexes are required to initiate neuronal cell responses, such as induced neurite outgrowth and cell differentiation [23]. Clathrin-coated membranes are involved in the endocytosis of Trk receptors [24], and therefore γ-enolase would be expected to be in these clathrin-coated vesicles. In the differentiated SH-SY5Y cells, the clathrin-coated vesicles were visualised using antibodies to clathrin heavy chain, under confocal fluorescence microscopy. In the wild-type SH-SY5Y cells, there was prominent co-localisation of γ-enolase with clathrin heavy chain at the plasma membrane (Fig. 2), whereas in the transfected SH-SY5Y cells with decreased γ1-syntrophin expression, there was little γ-enolase co-localisation to clathrin-coated vesicles (Fig. 2).

Lower levels of γ-enolase in clathrin-coated vesicles will most probably result in disrupted γ-enolase translocation towards the plasma membrane. Nevertheless, blocking clathrin-dependent endocytosis with the pharmacological inhibitor CPZ resulted in increased intensity of γ-enolase in clathrin-positive vesicles. This effect was seen around the peri-plasma membrane region and along neurites, and it was more evident after 2 h of CPZ treatment than after 24 h (Additional file 1: Figure S4). These data indicate that γ-enolase is involved in endosomal trafficking of transmembrane proteins, such as Trk.

### γ-Enolase mediates endosomal trafficking of Trk

**Fig. 3:**
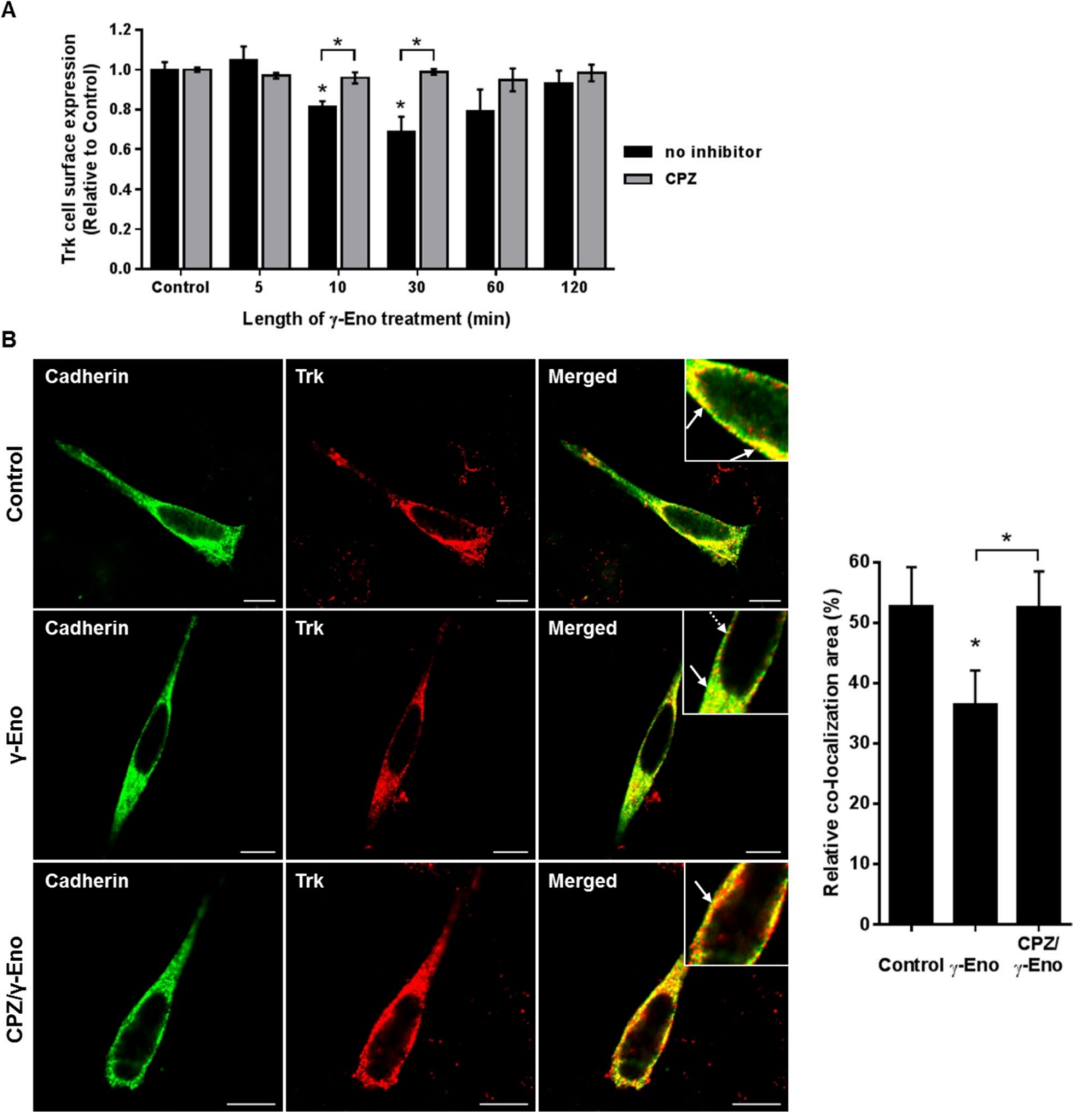
Effects of γ-Eno peptide on Trk cell-surface expression of SH-SY5Y cells. **A** Quantification from flow cytometric analysis of non-permeabilised cells for cell-surface expression of Trk as a time course of SH-SY5Y cells treated with 100 nM γ-Eno peptide in the absence and presence of 1 µM CPZ in serum-free medium. Data are means ± SD of three independent experiments (n = 3), each performed in duplicate (one-way ANOVA, Tukey’s test, *P < 0.05). **B** Representative images (left) and quantification (right) as relative co-localisation area of cadherin and Trk (solid arrows) of double immunofluorescence staining for the plasma membrane marker cadherin (green fluorescence) and Trk (red fluorescence) in SH-SY5Y cells pretreated with 1 µM CPZ for 30 min, followed by treatment with 100 nM γ-Eno peptide for an additional 30 min in serum-free medium. Data are means ± SD of the pixels in the third quadrant of the scatter plot (cell numbers ≥ 10) (one-way ANOVA, Tukey’s test, * *P* ˂ 0.05). Scale bars: 10 µm

To determine whether γ-enolase can interfere with internalisation of the Trk receptor, and consequently with its endosomal trafficking [25], we then examined the internalisation of cell surface Trk after exposure of the SH-SY5Y cells to the synthetic γ-enolase C-terminal peptide, γ-Eno peptide. Flow cytometry analysis showed that within 10 min of γ-Eno peptide treatment, cell surface Trk was significantly reduced. This effect was even more pronounced by 30 min, although after prolonged treatment, the cell surface levels of Trk returned to control levels (Fig. 3A). Additionally, treatment of SH-SY5Y cells with γ-Eno peptide increased level of phosphorylated Trk and the effect was most prominent as well after 30 min of peptide treatment (Additional file 1: Fig. 5A). However, inhibition of clathrin-dependent endocytosis with CPZ prior to γ-Eno peptide treatment prevented γ-Eno-peptide-mediated reduction in Trk levels at the cell surface of these SH-SY5Y cells. There were no changes in the cell surface Trk with CPZ alone (Fig. 3A). To ensure that the different levels of Trk at the cell surface after exposure to γ-Eno peptide reflect differences in Trk localisation rather than expression, γ-Eno-peptide-treated SH-SY5Y cells were permeabilised and then stained, to determine total Trk expression in the cells. After permeabilisation, there were no significant differences in Trk expression in the absence and presence of γ-Eno peptide, as determined by flow cytometry (Additional file 1: Figure S5).

A similar feature of Trk localisation to the plasma membrane region in response to γ-Eno peptide was observed by double immunofluorescence staining. In the control SH-SY5Y cells, Trk strongly co-localised with the plasma-membrane marker cadherin (Fig. 3B). However, in cells treated with γ-Eno peptide for 30 min, co-localisation of Trk with cadherin was weaker (Fig. 3B). In contrast, pre-exposure of the cells to CPZ followed by γ-Eno peptide showed the same co-localisation pattern of Trk with the plasma membrane marker as for the control cells (Fig. 3B). Thus, these data show that γ-Eno peptide treatment markedly influences Trk internalisation.

**Fig. 4:**
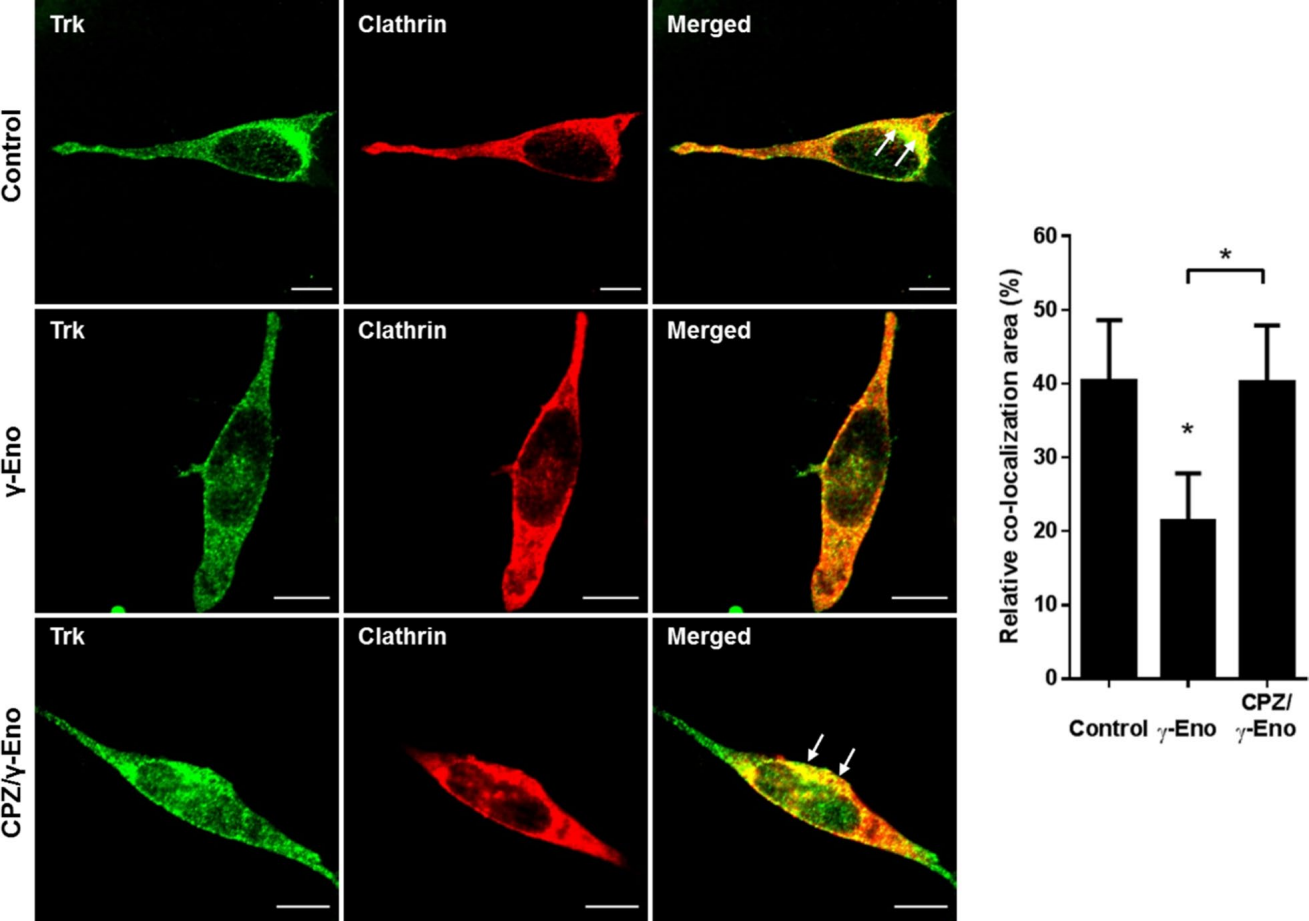
Effects of γ-Eno peptide on Trk localisation in clathrin-coated vesicles in SH-SY5Y cells. Representative images (left) and quantification (right) as relative co-localisation area of Trk and clathrin (solid arrows) of double immunofluorescence staining for Trk (green fluorescence) and clathrin heavy chain (red fluorescence) in SH-SY5Y cells pretreated with 1 µM CPZ for 2 h, followed by treatment with 100 nM γ-Eno peptide for an additional 30 min in serum-free medium. Data are means ± SD of the pixels in the third quadrant of the scatter plot (cell numbers ≥ 10) (one-way ANOVA, Tukey’s test, * *P* ˂ 0.05). Scale bars: 10 µm

This internalisation of cell surface Trk mediated by γ-Eno peptide suggested that γ-enolase interferes with Trk endosomal trafficking. Double immunofluorescence staining was used to localise Trk-positive organelles after γ-Eno peptide treatment. First, co-localisation of Trk with a marker of the clathrin-coated vesicles was examined. As expected, Trk was present in these vesicles in the peri-plasma membrane region in the differentiated SH-SY5Y cells (Fig. 4). Interestingly, γ-Eno peptide treatment resulted in significantly reduced co-localisation of Trk with clathrin heavy chain, with only a few clathrin-coated vesicles positive for Trk staining (Fig. 4). In contrast, pre-treatment of the differentiated SH-SY5Y cells with CPZ reversed the effects of γ-Eno peptide (Fig. 4).

**Fig. 5:**
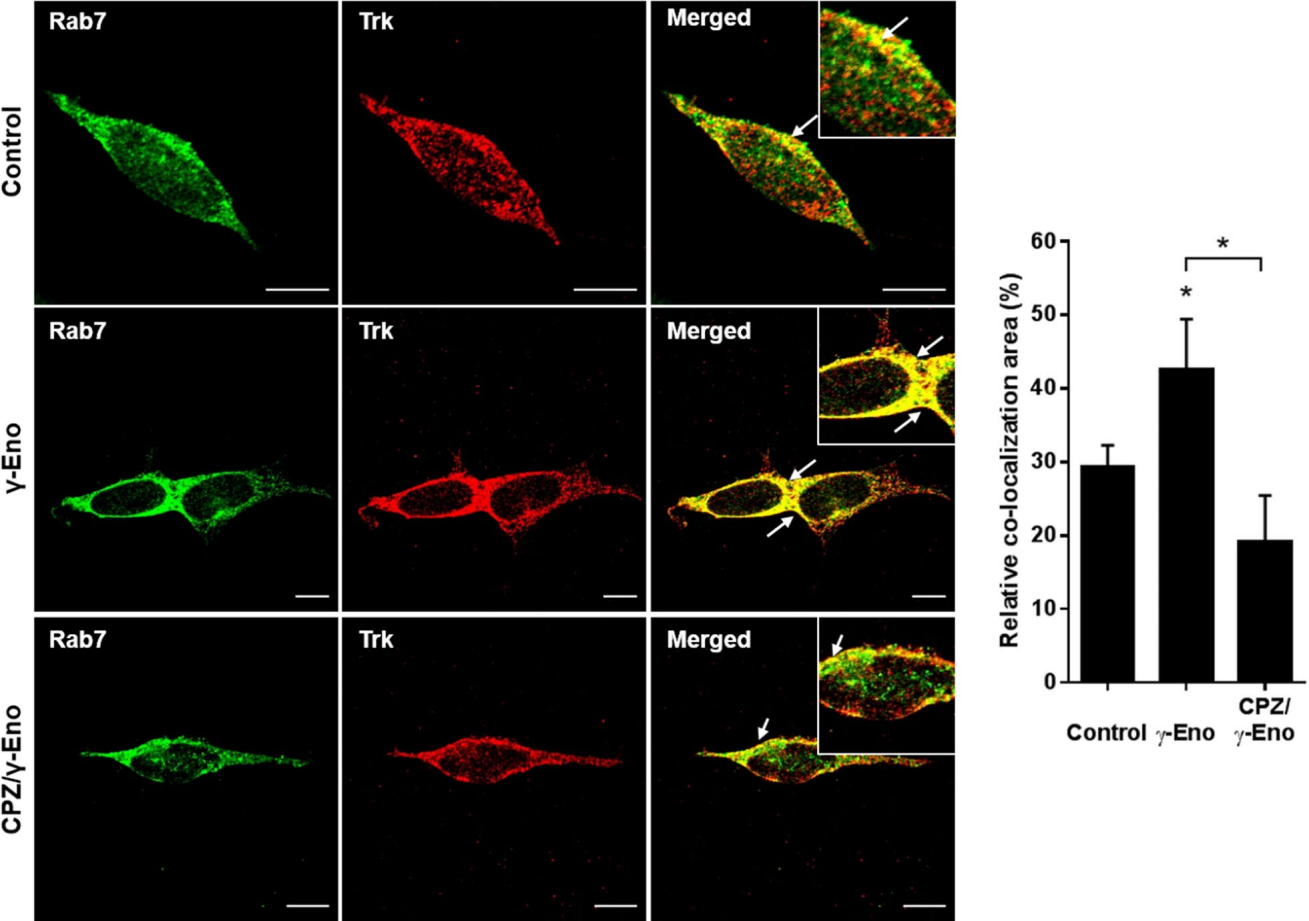
Effects of the CPZ on γ-enolase-mediated Trk endosomal localisation in SH-SY5Y cells. Representative images (left) and quantification (right) as relative co-localisation area of Rab7 and Trk (solid arrows) of double immunofluorescence staining for the marker for late endosomes Rab7 (green fluorescence) and Trk (red fluorescence) in SH-SY5Y cells pretreated with 1 µM CPZ for 2 h, followed by treatment with 100 nM γ-Eno peptide for an additional 30 min, in serum-free medium. Data are means ± SD of the pixels in the third quadrant of the scatter plot (cell numbers ≥ 10) (one-way ANOVA, Tukey’s test, * *P* ˂ 0.05). Scale bars: 10 µm

Based on these results, we next examined the trafficking of Trk receptor to late endosomes in response to γ-Eno peptide treatment using an antibody against Rab7. The immunofluorescence signal for the Trk receptor became concentrated with Rab7-positive late endosomes at 30 min after γ-Eno peptide treatment (Fig. 5), which correlated well with the data obtained with the marker for clathrin-coated vesicles (Fig. 4). Pre-exposure to CPZ resulted in significantly decreased transport of the Trk receptor to Rab7-positive late endosomes, as compared to γ-Eno peptide treatment alone, and co-localisation of Trk with Rab7 was weaker than that of control cells (Fig. 5). Altogether, these data confirm the role of γ-enolase in Trk trafficking and endocytic signalling.

**Fig. 6:**
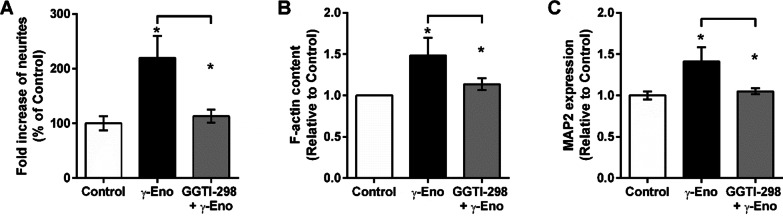
Effects of the Rap1 processing inhibitor GGTI-298 on γ-Eno peptide-mediated neurite outgrowth of SH-SY5Y cells. SH-SY5Y cells were pre-treated with 5 µM GGTI-298 for 1 h, followed by treatment with 100 nM γ-Eno peptide. **A** After 48 h of γ-Eno peptide treatment, neurite outgrowth was determined by counting neurites, where cells with neurites longer than the cell diameter were scored. Data are means ± SD of three independent experiments (n = 5), each performed in quadruplicate (one-way ANOVA, Tukey’s test, *P < 0.05). **B** After 2 h of γ-Eno peptide treatment, F-actin content was determined by flow cytometry following staining with 500 ng/mL TRITC-conjugated phalloidin. Data are means ± SD of three independent experiments (n = 3), each performed in duplicate (one-way ANOVA, Tukey’s test, *P < 0.05). **C** Flow cytometry analysis of After 24 h of γ-Eno peptide treatment, MAP2 expression was determined by immunostaining with a specific MAP2 antibody. Data are means ± SD of three independent experiments (n = 3), each performed in duplicate (one-way ANOVA, Tukey’s test, *P < 0.05)

Intracellular trafficking to late endosomes is required for Rap1 activation, which leads to neurite outgrowth [26]. To show a link between endosomal trafficking and γ-enolase neuritogenic activity, we pretreated SH-SY5Y cells with the inhibitor of Rap1 processing, GGTI-298, 1 h prior to γ-Eno peptide treatment, and evaluated neurite outgrowth. This pre-exposure to GGTI-298 led to significant reduction in the formation of neurite elongations mediated by γ-Eno peptide (Fig. 6A). Additionally, GGTI-298 also decreased the F-actin content (Fig. 6B) and reduced the protein levels of MAP2 (Fig. 6C), both of which were significantly increased in response to γ-Eno peptide treatment.

### Biological significance of Trk in γ-Eno-peptide-mediated neurotrophic activity

**Fig. 7:**
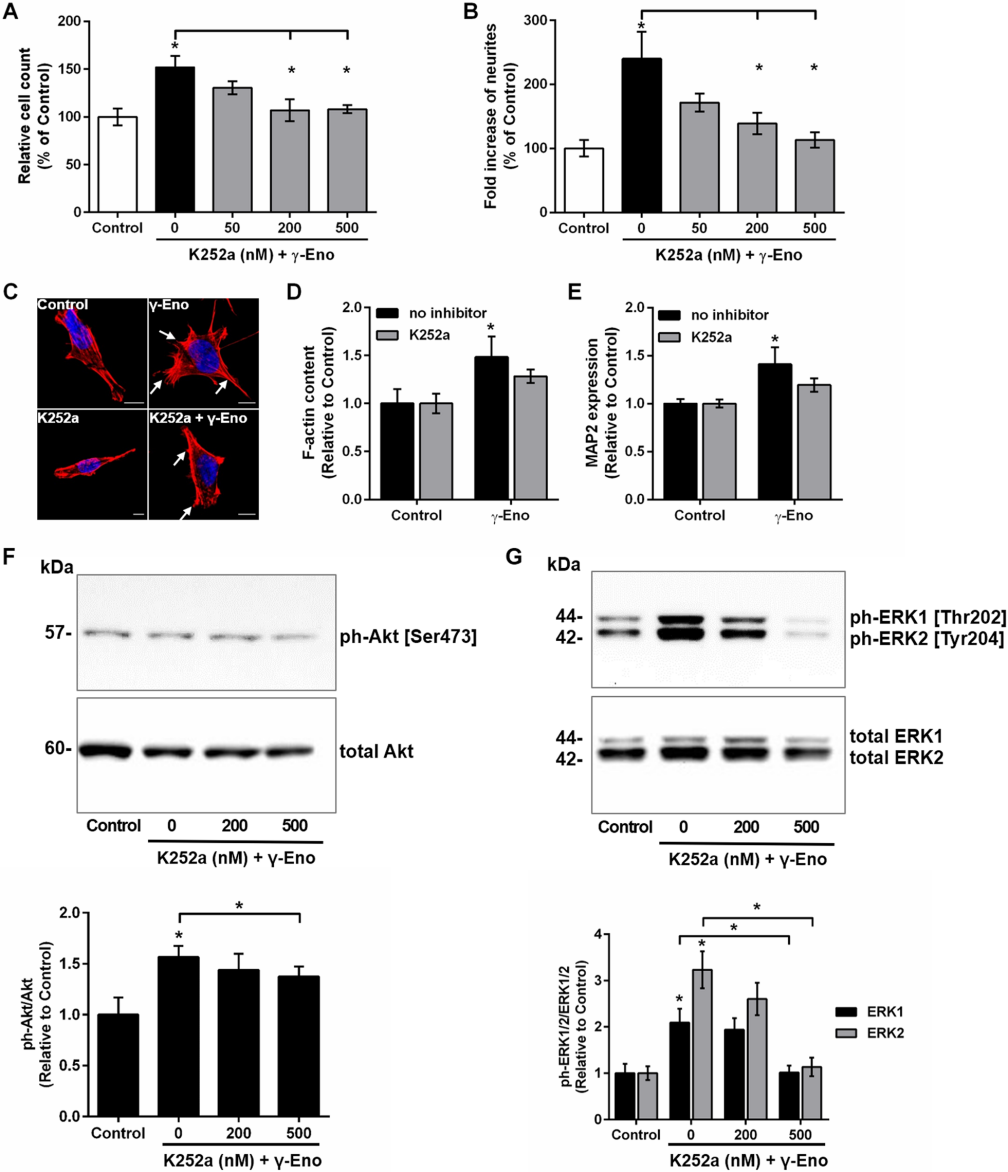
Effects of the tyrosine protein kinase activity inhibitor K252a on γ-Eno peptide-mediated cell survival and neurite outgrowth in SH-SY5Y cells. **A**, **B** SH-SY5Y cells were pretreated for 1 h with 50 nM to 500 nM K252a, the inhibitor of tyrosine protein kinase activity, followed by treatment with 100 nM γ-Eno peptide for 48 h. Quantification of cell survival using the MTS assay (**A**) and neurite outgrowth by counting the neurites (**B**), where cells with neurites longer than the cell diameter were scored. Data are means ± SD of three independent experiments (n = 3), each performed in quadruplicate (one-way ANOVA, Tukey’s test, *P < 0.05). **C**–**E** SH-SY5Y cells were treated with 100 nM γ-Eno peptide in the absence and presence of 200 nM K252a. **C**, **D** Representative images and quantification (as staining with 500 ng/mL TRITC-conjugated phalloidin) of fluorescence staining for actin filaments (solid arrows) by 500 ng/mL TRITC-conjugated phalloidin (red fluorescence) after 2 h of γ-Eno peptide treatment. Nuclei were counterstained with DAPI (blue fluorescence). Scale bars: 20 µm. Data are means ± SD of three independent experiments (n = 3), each performed in duplicate (one-way ANOVA, Tukey’s test, *P < 0.05). **E** Quantification of MAP2 expression after 24 h of γ-Eno peptide treatment by FACS analysis for immunostaining with a specific MAP2 antibody. Data are means ± SD of three independent experiments (n = 3), each performed in duplicate (one-way ANOVA, Tukey’s test, *P < 0.05). **F**, **G** SH-SY5Y cells were incubated for 1 h with inhibitor K252a (200 nM), followed by treatment with 100 nM γ-Eno peptide for additional 1 h. Representative Western blots of Akt (**F**), and ERK1/2 (**G**) activation. Total lysates (30 µg) were separated by SDS/PAGE and subjected to immunoblotting using the indicated antibodies. Graphs below indicate the relative values of phosphorylated forms compared to respective controls (total form of kinase) as means ± SD of two independent experiments (n = 2) (one-way ANOVA, Tukey’s test, *P < 0.05)

Finally, to highlight the importance of the Trk receptor in mediating the neurotrophic effects of γ-enolase, we examined the effects of γ-Eno peptide on survival of the SH-SY5Y cells in the presence of the Trk kinase activity inhibitor K252a. As shown in Fig. 7A, pre-exposure with K252a reduced γ-Eno-peptide-promoted cell survival in a concentration-dependent manner, with significant effects at 200 nM and 500 nM K252a. At the same concentrations, K252a pre-treatment also significantly abolished formation of neurites induced by γ-Eno peptide treatment (Fig. 7B). However, the inhibitor alone showed no ffect on cell viability and minimal effect on reduced neurite outgrowth of SH-SY5Y cells (Additional file 1: Figure S6). One of the earliest events in neurite outgrowth is reorganisation of the cytoskeleton, which includes rapid induction of actin polymerisation [27]. Here, pre-exposure to 200 nM K252a reduced γ-Eno-peptide-triggered induction of rapid actin polymerisation at the tips of forming neurites of the SH SY5Y cells (Fig. 7C). This was also confirmed by flow cytometric analysis of the F-actin content, where K252a attenuated γ-Eno-peptide-induced increases in F-actin (Fig. 7D). Moreover, the neuritogenic response to γ-Eno peptide was also evaluated by examination of MAP2 protein levels, a neuron-specific cytoskeletal protein that is enriched in neurites. SH-SY5Y cell treatments with γ-Eno peptide significantly increased MAP2 protein levels, whereas pre-exposure to K252a prevented this (Fig. 7E). Due to effect of K252a on γ-Eno-mediated neurotrophic activity, we additionally assessed the degree of activation of the PI3K/Akt and MAPK/extracellular signal-regulated kinase (ERK) signalling pathways that have been shown relevant for γ-enolase action. Pre-exposure of cells to the K252a inhibitor prior to γ-Eno treatment resulted in decreased γ-Eno peptide-induced Akt (Fig. 7F) and the effect of K252a was even more prominent in reduction of ERK1/2 phosphorylation (Fig. 7G).

**Fig. 8:**
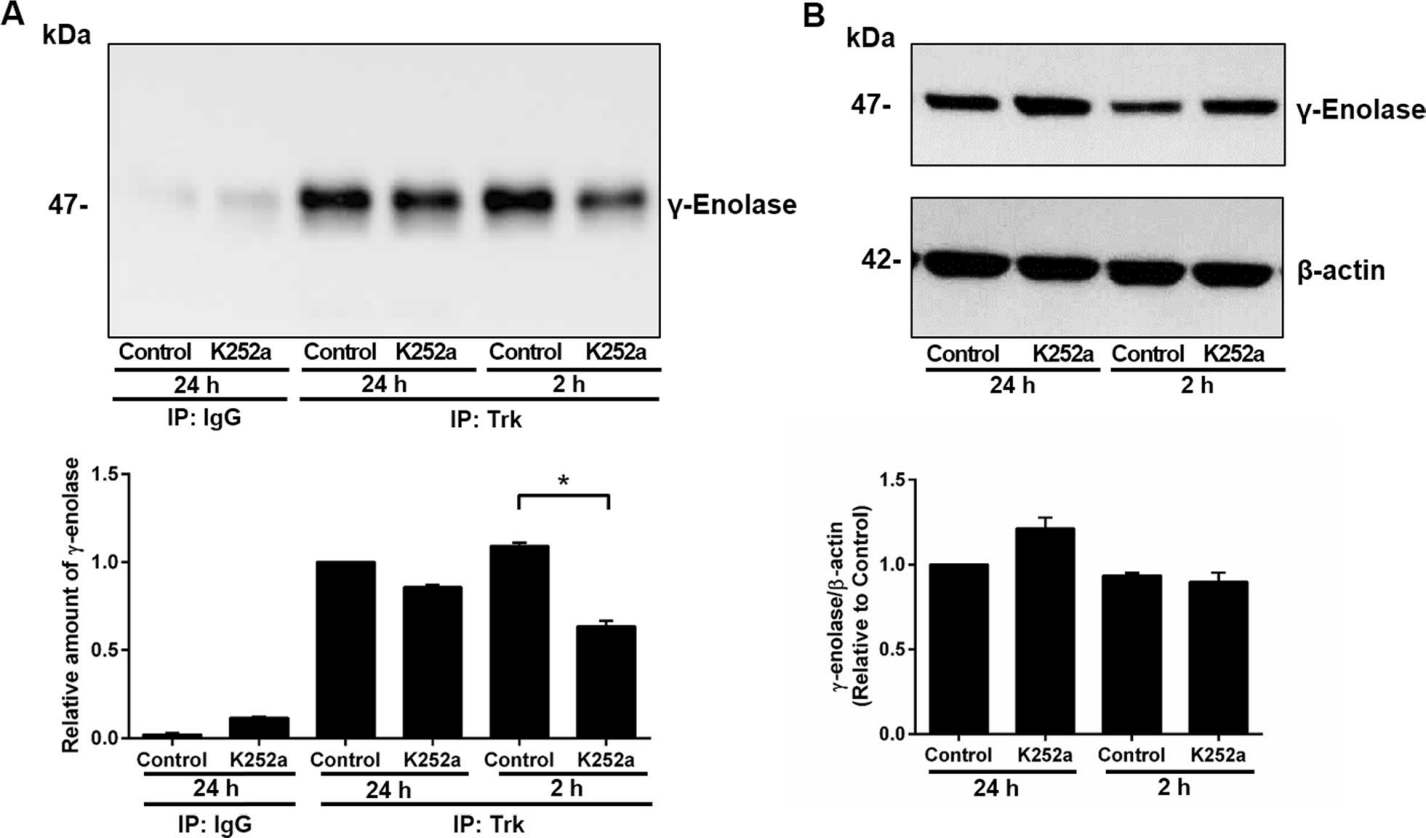
Effects of the tyrosine protein kinase activity inhibitor K252a on Trk/γ-enolase complex formation in SH-SY5Y cells. **A**, **B** Representative co-immunoprecipitation (top) and quantification (bottom; as relative amount of γ-enolase in the immunoprecipitate in treated cells vs. control cells at 24 h) of γ-enolase and Trk from SH-SY5Y cells treated with K252a (200 nM) for 2 h and 24 h. After treatments, cells were lysed and immunoprecipitated (IP) with either non-specific rabbit IgG or rabbit anti-*pan*-Trk antibodies (Trk). The immunoprecipitates (**A**) and lysates for immunoprecipitation (**B**) were analysed by Western blotting probed with a mouse anti-γ-enolase antibody. In case of lysates, β-actin was used as loading control. Data are means of two independent experiments (n = 2) (one-way ANOVA, Dunnett’s test, *P < 0.05)

Finally, we supported the significance of Trk activation in γ-enolase neurotrophic activity by determination of Trk/γ-enolase complex formation in SH-SY5Y cells with abolished Trk kinase activity. We performed additional immunoprecipitation experiments with K252a inhibitor. We co-immunoprecipitated Trk-γ-enolase complex from cell lysates of cells treated with K252a inhibitor, where we observed that the presence of the inhibitor reduced the association between Trk and γ-enolase. The significant effect in decreased amount of the complex was observed only at 2 h of K252a cell treatment, however, after 24 h of K252a treatment no such effect was observed (Fig. 8A), probably due to its lower concentration or effectiveness. Nevertheless, K252a inhibitor did not alter the expression of γ-enolase, indicating the dependence of the complex formation on the Trk phosphorylation rate. Taken together, research findings suggest that the kinase activity of Trk is required for γ-enolase-mediated neurotrophic signalling.

## Discussion

The purpose of this study was to investigate the interactions of γ-enolase with neurotrophin receptors at the plasma membrane in SH-SY5Y cells, and to define the significance of these interactions. The intracellular trafficking of cytosolic γ-enolase towards the plasma membrane, a step pre-requisite for its neurotrophic activity, is known to be regulated by scaffold protein γ1-syntrophin [14]; however, the association of γ-enolase with the membrane receptor or other binding partners at the plasma membrane remains unknown.

Here, we show that γ-enolase associates with the intracellular domain of the Trk receptor in differentiated SH-SY5Y cells, and that this interaction mainly occurs in the peri-plasma membrane region. Shortly after serum deprivation, these neuroblastoma cells flatten and produce filopodia and lamellipodia around their circumference. Over the subsequent 16 h to 24 h, the cells gradually extend neurites [28, 29], within which γ-enolase is highly concentrated [14]. On the other hand, relatively weak co-localisation is seen for γ-enolase with the other neurotrophin receptor, p75^NTR^, and this was not seen at the plasma membrane in either the control or the differentiated SH-SY5Y cells. This is in line with our previous study, where there were no significant interactions of γ-enolase with p75^NTR^ in control PC12 cells, whereas exposure of these cells to the toxic amyloid-β-peptide that leads to apoptosis resulted in strong association of γ-enolase and p75^NTR^ in the peri-plasmic region of injured PC12 cells [21]. Nevertheless, in differentiated SH-SY5Y cells where γ1-syntrophin-guided translocation of γ-enolase towards the plasma membrane is disrupted, the association of γ-enolase with Trk was less prominent. Additionally, in these cells, co-localisation of γ-enolase with the intracellular domain of the Trk receptor was abolished, which suggests that under non-degenerative conditions, γ1-syntrophin-translocated γ-enolase is implicated in coupling with the tyrosine kinase receptor, or with its associated signalling complex that is required for neurotrophic signalling.

A further goal of this study was to define the relative significance of the Trk receptor in cell survival and neurite outgrowth promoted by γ-enolase. NGF, the prototype neurotrophin, elicits most of its neurotrophic effects by binding and activating TrkA, which causes dimerisation of this Trk receptor and activation of its kinase domain p140^prototrk^, with the consequent activation of well-defined downstream signalling cascades [17, 24]. This action upon NGF-specific signal transduction is blocked by the Trk kinase inhibitor K252a, reversible cell-permeable inhibitor, which impairs tyrosine phosphorylation and the kinase activity of p140^prototrk^ in nanomolar range [30]. Pre-exposure of SH-SY5Y cells to K252a blocked the effects of γ-Eno peptide in terms of its neurotrophic actions, and promoted cell survival and increased neurite outgrowth. K252a reduced γ-Eno-peptide-promoted cell survival in a concentration-dependent manner. At the same concentrations, K252a pre-treatment significantly abolished the formation of neurites induced by γ-Eno peptide treatment. One of the earliest events in neurite outgrowth is the reorganisation of the cytoskeleton, which includes rapid induction of actin polymerisation [27]. As observed, the K252a inhibitor reduced γ-Eno-peptide-triggered induction of rapid actin polymerisation at the tips of the forming neurites of these SH SY5Y cells, and consequently increased γ-Eno-peptide-induced protein levels of MAP2, a neuron-specific cytoskeletal protein that is a well-known marker for neurite outgrowth [31]. These data are in line with the effects of K252a on the neuritogenic activities of other neurotrophins. It has been shown that K252a inhibits NGF-elicited generation of neurites [32]. Additionally, association between γ-enolase and Trk depended on the tyrosine phosphorylation of Trk as we observed reduced level of Trk/γ-enolase complex formation in cells exposed to inhibitor of Trk kinase activity, K252a. Mode of K252a action toward the reduced amount of the complex was observed at 24 h, however, the significant effect in reduced association between Trk and γ-enolase was observed only at 2 h of K252a treatment, probably due to its lower concentration or effectiveness. Based on the results obtained with immunoprecipitation method and functional assays evaluating the neuritogenic effects mediated by γ-Eno peptide we suggest that association between Trk and γ-enolase in SH-SY5Y cells is present and that this association is indeed dependent on tyrosine kinase activity of the receptor as K252a inhibitor diminishes but not completely abolishes the association probably due to the reversible mode of K252a inhibitor action. The latter is in line with the results of cell viability, neurite outgrowth assay and F-actin content analysis that also showed that K52a inhibitor did not completely abolished the neuritogenic effect of γ-Eno peptide on SH-SY5Y cells.

There is increasing evidence that internalisation and transport of the neurotrophin receptor complex are required to initiate cell responses [33, 34]. Ehlers et al. [35] showed that NGF induces rapid and extensive endocytosis of TrkA in distal dorsal root ganglion neurons. Additionally, Zhang et al. [23] demonstrated that distinct biological responses to NGF are controlled by receptor signalling from different locations within the cells. Survival responses to NGF are only initiated by activated receptors at the cell surface, where they orchestrate prolonged activation of the kinase Akt. On the contrary, neurite outgrowth and neuronal differentiation are promoted by Trk receptor internalisation, in addition to receptor activation at the cell surface. Moreover, Yu et al. [36] showed that regulation of trafficking of activated TrkA is critical for NGF-mediated functions. In the present study, we used two different methods to define the disposition of the Trk cell-surface receptors on these SH-SY5Y cells. Here, internalisation of Trk was increased after exposure of the cells to γ-Eno peptide. Indeed, there were significant decreases in Trk on the cell surface at the shorter times of this γ-Eno peptide treatment, with the most prominent effects seen after 30 min treatment. This indicates that γ-enolase is involved in the intracellular trafficking of Trk.

Studies performed both in vitro and in vivo have demonstrated that neurotrophin receptors internalised into vesicles remain activated for as long as the ligand remains associated with the receptor [36–39]. The Trk receptor within the FRS2-Crk-C3G complex is internalised into clathrin-coated vesicles and transported through early to late endosomes [40], where Rap1 is activated, which leads to activation of the B-Raf–MEK–ERK pathway. As we showed in our recent study, γ-enolase can only induce activation of B-Raf kinase, and not of c-Raf kinase [6], which suggests a signalling pathway where clathrin-mediated endocytosis is required. Indeed, in serum-depleted SH-SY5Y cells, γ-enolase co-localised with the clathrin heavy chain, whereas in cells with down-regulated γ1-syntrophin, γ-enolase failed to co-localise with clathrin-coated vesicles, which indicates that γ-enolase is part of the vesicular trafficking. Blocking clathrin-dependent endocytosis with CPZ prior to γ-Eno peptide treatment prevented γ-Eno-peptide-mediated reduction of Trk levels at the cell surface of these SH-SY5Y cells, as well as co-localisation of Trk with the plasma membrane marker cadherin. However, γ-Eno peptide triggered phosphorylation of Trk in a time-dependent manner, indicating on the ability of γ-Eno peptide to promote Trk receptor activity. Furthermore, γ-Eno peptide treatment resulted in significantly reduced co-localisation of Trk with clathrin heavy chain, where only a few clathrin-coated vesicles were positive for Trk staining. In contrast, pre-exposure to CPZ reversed the effects of γ-Eno peptide, which resulted in strong localisation of Trk with clathrin heavy chain, thus confirming the role of γ-enolase in endosomal trafficking of Trk.

Internalisation and vesicular trafficking of the TrkA receptor from clathrin-coated vesicles [24, 41], through early endosomes [42], to late endosomes is essential for sustained activation of Rap1 kinase, which leads to increased neurite outgrowth upon NGF stimulation [26]. Consistent with these observations, immunofluorescence confocal microscopy revealed that the signal for Trk at Rab7-positive late endosomes was greatly increased after γ-Eno peptide treatment, whereas block of clathrin-mediated endocytosis completely abolished co-localisation of Trk with Rab7, a marker for late endosomes. Nevertheless, the co-localisation correlated well with Trk in clathrin-coated vesicles as a result of γ-Eno peptide treatment, which suggests that γ-enolase indeed interferes in the endosomal trafficking of Trk. Nevertheless, we assume that γ-enolase-mediated Trk endosomal trafficking occurs at the peri-plasma membrane region within the cells. This is in line with a recent study that proposed a model for Trk activation in which the Trk receptors have a propensity to interact laterally and to form dimers even in the absence of ligand [43].

Intracellular, clathrin-mediated endocytosis of Trk and its trafficking to late endosomes is required for Rap1 activation and sustained activation of ERKs, which leads to neurite outgrowth [26, 44]. Similarly, in our previous study, sustained ERK activation by γ-Eno peptide was shown to be necessary for neurite outgrowth of SH-SY5y cells [6] and here we have shown that γ-Eno induced ERK activation was abolished in cell with reduced Trk kinase activity achieved by K252a inhibitor. Therefore, we suppressed Rap1 function here by treating these SH-SY5Y cells with the Rap1 processing inhibitor GGTI-298, which specifically inhibits Rap1 but not Ras GTPase [45, 46]. This demonstrated that inhibition of Rap1 prevents γ-Eno-peptide-mediated neuritogenic effects, as observed by disrupted rapid actin polymerisation and the consequent reduced formation of extended neurites and neurite outgrowth. This further supports the need for γ-enolase endosomal trafficking for neurite outgrowth.

## Conclusions

**Fig. 9:**
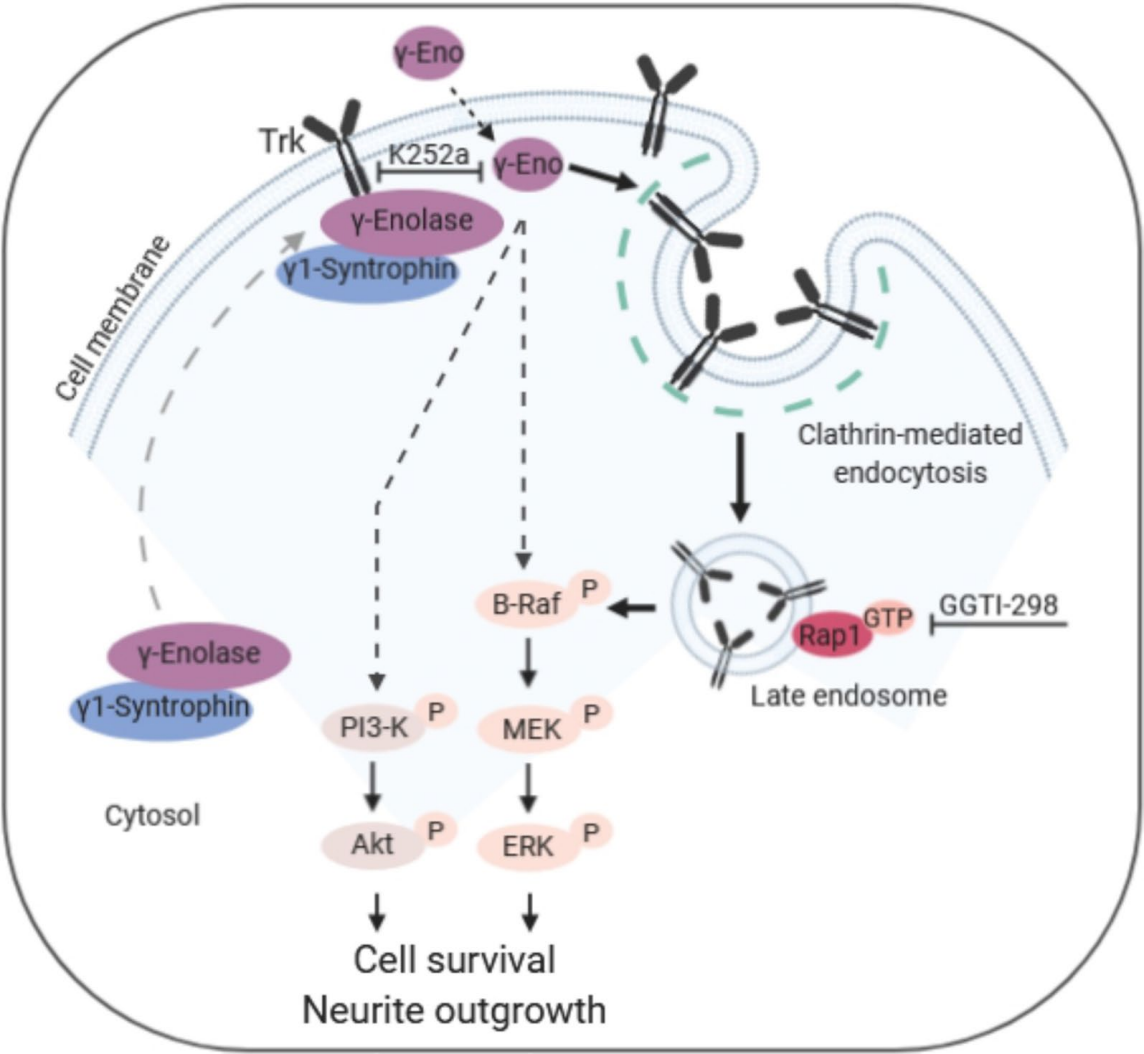
Model of Trk trafficking under γ-enolase-induced neurotrophic signalling. In differentiated SH-SY5Y cells, γ-enolase is translocated to the plasma membrane with γ1-syntrophin, where it associates with the intracellular domain of the Trk receptor. Treating differentiated SH-SY5Y cells with the biological active C-terminal peptide of γ-enolase (γ-Eno peptide) triggers Trk receptor internalisation by clathrin-mediated endocytosis, and thence its trafficking to late endosomes, which leads to Rap1 activation. As well as γ-Eno-peptide-mediated activation of the PI3-K/Akt and MAPK/ERK signalling pathways, these signals are required for γ-Eno-peptide-induced cell survival and neurite outgrowth

To date, γ-enolase has been described as a neurotrophic-like factor that controls neuronal survival and neurite outgrowth through activation of the PI3K/Akt and MAPK/ERK signalling pathways [6]. Herein, we provide the first evidence that γ-enolase-mediated neurotrophic activity requires activation of the neurotrophin Trk receptor, triggering activation of PI3K/Akt and MAPK/ERK signalling pathways. Scaffold protein γ1-syntrophin provides the trafficking of γ-enolase to specialised plasma membrane domains, where γ-enolase associates with the intracellular domain of Trk. As proposed in the overall scheme illustrated in Fig. 9, γ-enolase is an integral part of the signal transduction complex in neuronal cells that promotes Trk receptor activation through internalisation and endosomal trafficking, to elicited neurite outgrowth.

## Supplementary Information

**Additional file 1.** Supplementary Figures.

## Data Availability

All data generated or analyzed during this study are included in this published article [and its supplementary information files]. The datasets used and/or analyzed during the current study are also available from the corresponding author.

## Abbreviations

BDNF: Brain-derived neurotrophic factor
BSA: Bovine serum albumin
ERKs: Extracellular signal-regulated kinases
MAPK: Mitogen-activated protein kinase
MTS: 3-(4,5-Dimethylthiazol-2-yl)-5-(3-carboxymethoxyphenyl)-2-(4-sulfophenyl)-2H-tetrazolium
NGF: Nerve growth factor
NT-3: Neurotrophin-3
NT-4/5: Neurotrophin-4/5
p75^NTR^: Neurotrophin receptor p75
PBS: Phosphate-buffered saline
PI3K: Phosphatidylinositol 3-kinase
SD: Standard deviation
TRITC: Tetramethylrhodamine B isothiocyanate
Trk: Tropomyosin receptor kinase
