# Supplementary material for: γ-Enolase enhances Trk endosomal trafficking and promotes neurite outgrowth in differentiated SH-SY5Y cells
Source: Cell Commun Signal. 2021 Dec 11;19:118. doi: 10.1186/s12964-021-00784-1 (PMC8665614; doi:10.1186/s12964-021-00784-1)
Supplement: Supplementary file 2 — Additional file 1. Supplementary Figures. [file 12964_2021_784_MOESM2_ESM.docx]

**SUPPLEMENTAL INFORMATION**

# γ-Enolase enhances Trk endosomal trafficking and promotes neurite outgrowth in differentiated SH-SY5Y cells

**[Trk endosomal trafficking mediated by γ-enolase]**

**Anja Pišlar**^1,^* and **Janko Kos**^1,2^

^1^ Department of Pharmaceutical Biology, Faculty of Pharmacy, University of Ljubljana, Aškerčeva 7, 1000 Ljubljana, Slovenia, ^2^ Department of Biotechnology, Jožef Stefan Institute, Jamova 39, 1000 Ljubljana, Slovenia

*** Correspondence to: Anja Pišlar**

Faculty of Pharmacy, University of Ljubljana, Aškerčeva 7, 1000 Ljubljana, Slovenia, Tel: +386-1-4769526; Fax: +386-1-4258031; E-mail: [anja.pislar@ffa.uni-lj.si](mailto:anja.pislar@ffa.uni-lj.si)


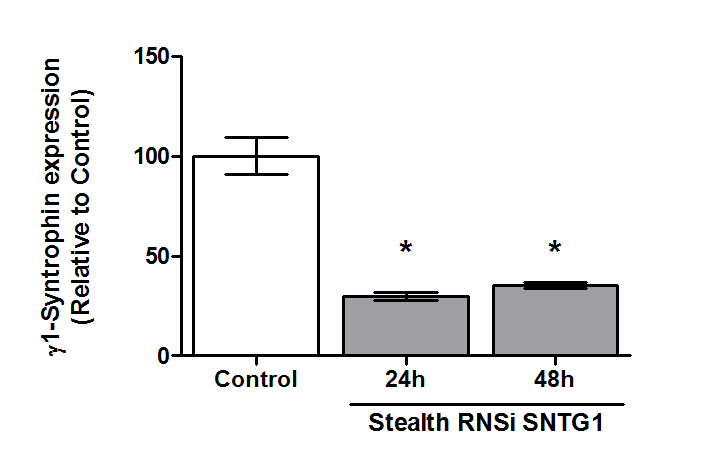


**Supplemental Figure S1.** γ1-Syntrophin protein levels in wild-type and transfected SH-SY5Y cells. Quantification of flow cytometry analysis of γ1-syntrophin expression 24 h and 48 h after transfection of SH-SY5Y cells with StealthTM RNAi/γ1-Syn, using immunostaining with a specific γ1-syntrophin antibody. Data are means ±SD of at least two independent experiments (n = 2), each performed in duplicate (one-way ANOVA, Dunnett’s test, **P* < 0.05).

**
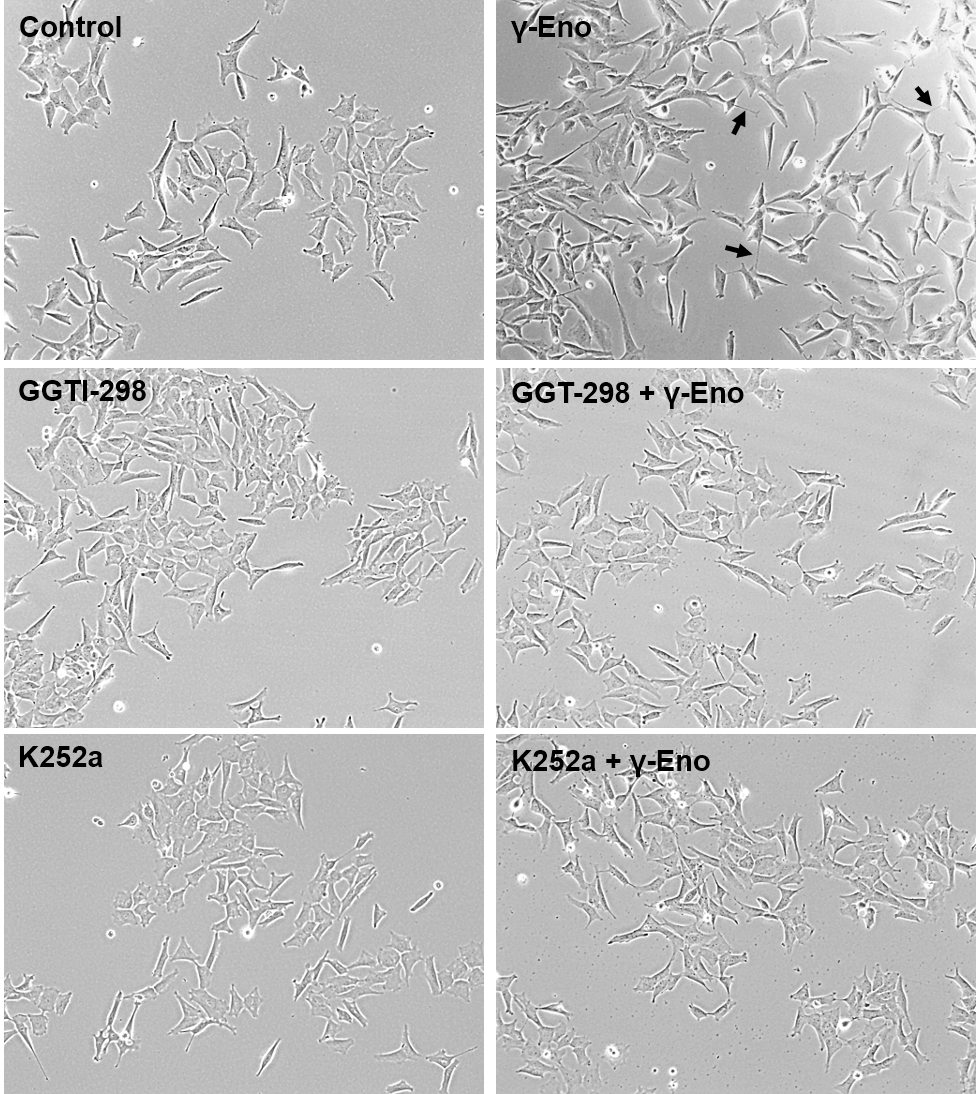
**

**Supplemental Figure S2.** Effect of K252a and GGTI-298 inhibitors on γ-Eno-mediated neurite outgrowth. Representative images of morphological changes in SH-SY5Y cells 48 h after exposure to γ-Eno (100 nM) alone or in presence of K252a (200 nM) or GGTI-298 (5 µM) inhibitors.


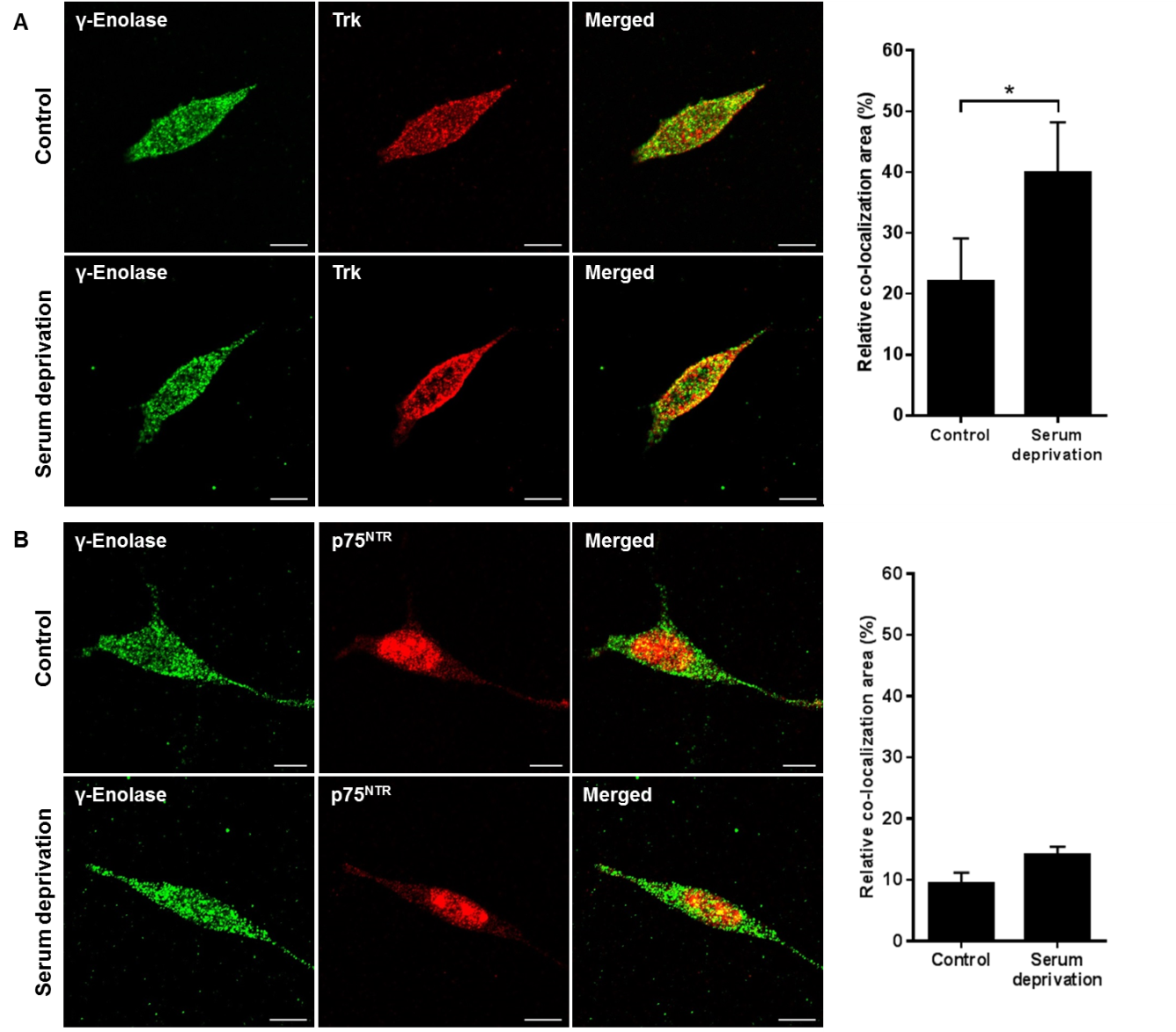


**Supplemental Figure S3.** γ-Enolase co-localisation with endogenous neurotrophin receptors in SH-SY5Y cells. (**A, B**) Representative images (left) and quantification (right; as relative co-localization area of γ-enolase and neurotrophin receptor) of double immunofluorescence staining for γ-enolase (green fluorescence) and endogenous neurotrophin receptors (red fluorescence), as *pan*-Trk (**A**) and p75^NTR^ (**B**), in control SH-SY5Y cells and SH-SY5Y cells differentiated by serum deprivation for 24 h. Data are means ±SD of the pixels in the third quadrant of the scatter plot (cell numbers ≥10) (two-tailed *t* test* *P* ˂ 0.05). Scale bars: 10 µm.


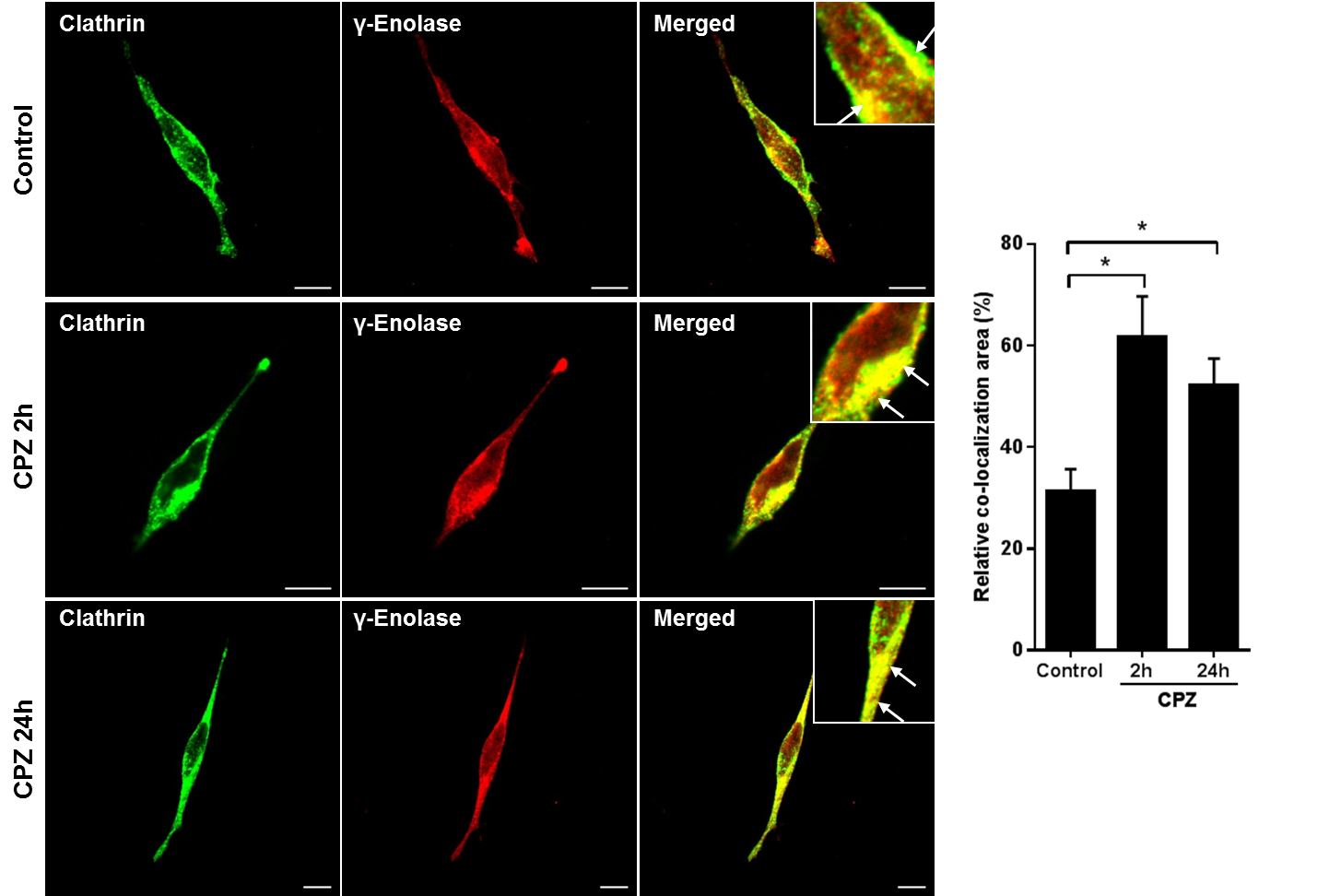


**Supplemental Figure S4.** Effects of CPZ on γ-enolase localisation in clathrin-coated vesicles in SH-SY5Y cells. Representative images (left) and quantification (right; as relative co-localisation area of clathrin and γ-enolase) of double immunofluorescence staining for clathrin heavy chain (green fluorescence) and γ-enolase (red fluorescence) in SH-SY5Y cells exposed to 1 µM CPZ for 2 h (CPZ 2h) and 24 h (CPZ 24h) in serum-free medium. SH-SY5Y cells treated with vehicle (dimethylsulphoxide) define the control. Data are means ±SD of the pixels in the third quadrant of the scatter plot (cell numbers ≥10) (one-way ANOVA, Tukey’s test, * *P* ˂ 0.05). Scale bars: 10 µm.


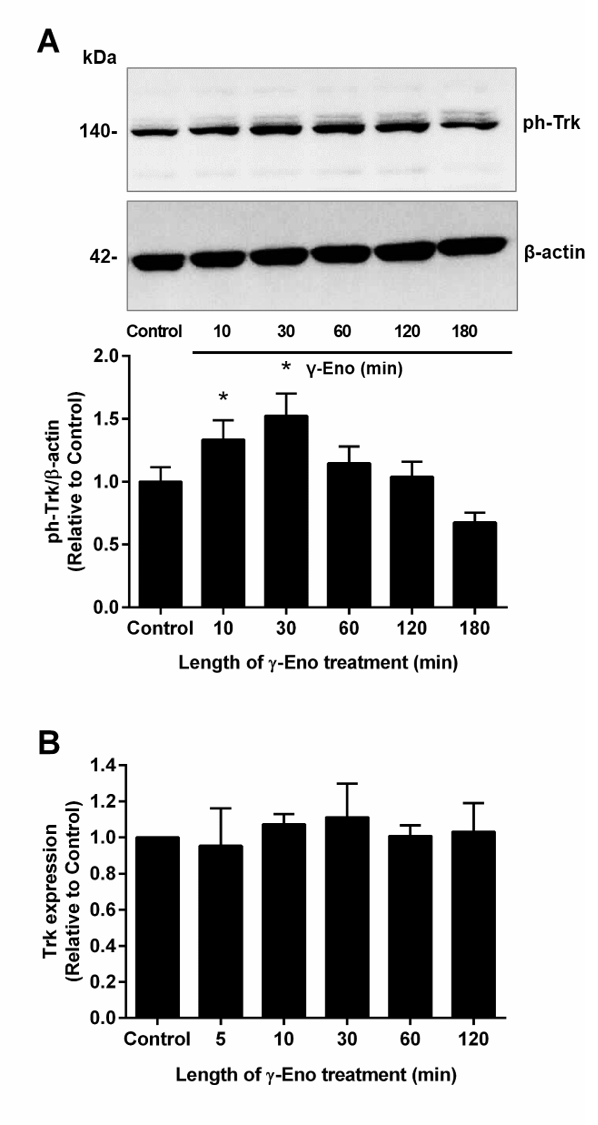


**Supplemental Figure S5.** Effects of the γ-Eno peptide on Trk phosphorylation and protein levels and in SH-SY5Y cells. (**A**) SH-SY5Y cells were treated with 100 nM γ-Eno peptide for 10, 30, 60, 120 and 180 min in serum-free medium, followed by western blot analysis of level of phosphorylated form of Trk with concomitant β-actin protein level as loading control. Data are means of two (n = 2) independent experiments. (one-way ANOVA, Dunnett’s test, **P* <0.05). (**B**) SH-SY5Y cells were treated with 100 nM γ-Eno peptide for 5, 10, 30, 60, and 120 min in serum-free medium, followed by flow cytometric analysis of permeabilised cells for expression of Trk. Data are means ±SD of three independent experiments (n = 3), each performed in duplicate (one-way ANOVA, ns).





**Supplemental Figure S6.** Effects of the tyrosine protein kinase activity inhibitor K252a cell survival and neurite outgrowth in SH-SY5Y cells. (**A, B**) SH-SY5Y cells were treated with 50 nM to 500 nM K252a, the inhibitor of tyrosine protein kinase activity for 48 h, followed by the quantification of cell survival using the MTS assay (**A**) and neurite outgrowth by counting the neurites (**B**), where cells with neurites longer than the cell diameter were scored. Data are means ±SD of three independent experiments (n = 2), each performed in quadruplicate (onw-way ANOVA, ns).
